# Infection of *Phytophthora palmivora* Isolates on *Arabidopsis thaliana*

**DOI:** 10.3390/jof10070446

**Published:** 2024-06-26

**Authors:** Mariandrea García-Gaona, Hernán Mauricio Romero

**Affiliations:** 1 Biology and Breeding Research Program, Colombian Oil Palm Research Center, Cenipalma, Calle 98 No. 70-91, Piso 14, Bogota 111121, Colombia; mgarcia@cenipalma.org; 2 Department of Biology, Universidad Nacional de Colombia, Bogota 111321, Colombia

**Keywords:** pathosystem, oil palm, molecular interaction, effectors, bud rot

## Abstract

*Phytophthora palmivora*, a hemibiotrophic oomycete, causes diseases in several economically important tropical crops, such as oil palm, which it is responsible for a devastating disease called bud rot (BR). Despite recent progress in understanding host resistance and virulence mechanisms, many aspects remain unknown in *P. palmivora* isolates from oil palm. Model pathosystems are useful for understanding the molecular interactions between pathogens and hosts. In this study, we utilized detached leaves and whole seedlings of *Arabidopsis thaliana* Col-0 to describe and evaluate the infection process of three *P. palmivora* isolates (CPPhZC-05, CPPhZC-04, CPPhZOC-01) that cause BR in oil palm. Two compatible isolates (CPPhZC-05 and CPPhZOC-01) induced aqueous lesions at 72 h post-inoculation (hpi), with microscopic visualization revealing zoospore encysting and appressorium penetration at 3 hpi, followed by sporangia generation at 72 hpi. In contrast, an incompatible isolate (CPPhZC-04) exhibited cysts that could not penetrate tissue, resulting in low leaf colonization. Gene expression of ten *P. palmivora* infection-related genes was quantified by RT-qPCR, revealing overexpression in compatible isolates, but not in the incompatible isolate. Additionally, key genes associated with salicylic acid (SA), jasmonic acid (JA), and ethylene (ET) in Arabidopsis exhibited regulation during interaction with the three isolates. These findings demonstrate that *P. palmivora* can infect Arabidopsis Col-0, and variability is observed in the interaction between Arabidopsis-Col-0 and *P. palmivora* isolates. Establishing this pathosystem is expected to enhance our understanding of *P. palmivora*’s pathology and physiology.

## 1. Introduction

*Phytophthora palmivora* is a generalist pathogen that causes root, bud, and fruit rot diseases in various economically important tropical crops, including durian, papaya, coconut, rubber, and cocoa [1,2]. It is especially devastating in oil palm [3], causing bud rot disease [2,4], the main limiting factor for crop expansion in Latin America. *P. palmivora* is an oomycete characterized by having a hemibiotrophic life cycle. Like many oomycetes, it has an asexual life cycle, where sporangia capable of dispersing through wind or water are generated. These structures can germinate directly or by releasing zoospores [5].

In addition to the different agricultural and biological alternatives to mitigate *P. palmivora* infections in oil palm [6,7], studying the interaction between plants and pathogens provides a comprehensive overview of the defense mechanisms of the plant and pathogen infection. This knowledge can help to develop durable, effective, and environmentally friendly strategies [8].

Establishing model pathosystems in controlled experimental setups that mimic natural interactions is central to advancing our understanding of these interactions. Among the available model organisms, *Arabidopsis thaliana* stands out due to its genetic tractability, well-documented genome, and ease of transformation. Over time, Arabidopsis has become a favored choice for elucidating the complexities of host–pathogen interplay [9,10].

Within the genus *Phytophthora*, the infection processes of several species have been elucidated in *A. thaliana*. These species include *Phytophthora brassicae* [11], *P. parasítica* [12], *P. capsici* [13], *P. cinnamomi* [14], and *P. palmivora* [9,15]. Despite this progress, specific insights into the infection processes of different *P. palmivora* isolates remain unexplored, particularly in the context of economically relevant hosts like oil palm.

The aims of the study were (i) to elucidate the infection processes of three *P. palmivora* isolates in their interactions with *Arabidopsis thaliana*, (ii) to distinguish between compatible and incompatible interactions by analyzing the microscopic stages of infection, and (iii) to characterize the gene expression profiles of *P. palmivora’s* candidate effectors and the defense response markers in *A. thaliana*.

## 2. Materials and Methods

### 2.1. Plant Material

*Arabidopsis thaliana* ecotype Col-0 (CS667) was obtained from the ABRC collection. Maintenance of seeds and plant culture conditions followed a standard protocol [16]. Plants grew in pots with a mix of Floragard^®^ (Oldenburg, Germany) peat moss and perlite in a growth chamber at 22 °C, under a photoperiod of 10 h of light and 14 h of darkness, with a relative humidity of 60–70%.

### 2.2. Pathogen Growth and Infection Assay

Three *Phytophthora palmivora* isolates (CPPhZC-05, CPPhZC-04, CPPhZOC-01) from the Cenipalma collection which had been previously characterized [17] were grown on clarified V8 juice medium consisting of 20% V8^®^ Campbell (Camden, NJ, USA) juice, 5 g L^−1^ CaCO_3_ (Merck, Darmstadt, Germany), 50 mg L^−1^ B-sitosterol (Merck), 1.5% Bacto™ agar, and rifampicin 1 μg mL^−1^ (Merck), as previously reported [18]. The cultures were maintained under a 12 h photoperiod at 25 °C until 8 days of growth had been completed.

The detached leaf inculation was performed by following Wang et al. [12]. Twenty fully expanded leaves (leaf No 3, 4, or 5 of the rosette) from 4-week-old (growth stage 1.10–1.12) Col-0 plants were excised and placed onto moist filter paper in a tray (22 × 14 × 7 cm), then introduced into a Ziploc^®^ bag (San Diego, CA, USA). Zoospore suspensions of *P. palmivora* isolates (9 × 10^5^ zoospores/mL) were prepared, and a droplet of 10 µL was applied to the abaxial side of the leaf; sterile water was used as a control (mock inoculation). The release of zoospores was induced by thermal shock using sterile distilled water at 4 °C, and pathogen quantification was performed using a Neubauer Chamber. The inoculated trays were maintained at 25 °C with a 12 h light/dark photoperiod. 

Twenty detached leaves per treatment (CPPhZC-05, CPPhZC-04, CPPhZOC-01 and Mock) were scanned at 72 hpi. The total area of each leaf and the infected area were calculated using *Assess 2.0* [19]. Ten independent replicates were performed. 

For whole plant infection, we used the methodology described by Wang et al. [12] with modifications. Ten-day-old Arabidopsis seedlings (growth stage: 1.04) were floated in a suspension of 9 × 10^5^ zoospores mL^−1^ for five seconds and then transferred onto Petri plates containing half-strength MS medium without sugar. A total of 60 plants per each treatment, distributed in 10 petri plates, were used for inoculation. Then, the plates were incubated at 23 °C with a 12 h light/dark photoperiod for 96 h.

### 2.3. Microscopic Visualization of the Infection Process

Leaves or whole seedlings of *A. thaliana* were inoculated as decribed above. Samples for each treatment were collected at 3, 6, 24, 48, and 72 hpi. They were stained using diluted trypan blue solution of 10 g phenol (Merck), 10 mL glycerol (Promega, Madison, WI, USA), 10 mL lactic acid (Merck), 10 mL destiled water, and 10 mg trypan blue (Merck) [20]. Subsequently, the samples were destained in 96% ethanol for 48 h, replacing the ethanol every 12 h until the leaves cleared. Observations were made using an Olympus (Tokyo, Japan) CX3 microscope. Three independent replicates were conducted. 

For SEM visualization, three leaves from each treatment were fixed in 2.5% glutaraldehyde for 24 h and sequentially dehydrated in ethanol concentrations of 30%, 50%, 70%, 80%, 90%, and 95% for 10 min each, then finally at 100% overnight. The samples were then dried, coated with gold [4], and prepared for imaging.

### 2.4. Extraction of Total RNA, cDNA Synthesis, and qRT-PCR Analysis

After inoculation, ten leaves were collected at each time point, at 3, 6, 24, 48, and 72 hpi, then were homogenized with liquid nitrogen and stored at −80 °C. The extraction of RNA from the homogenized leaves for each treatment was carried out with the InviTrap^®^ Spin Plant RNA Mini Kit (Invitek, Tondela, Portugal) following the manufacturer’s protocol. The cDNA synthesis was performed using 1 ng of RNA with the SuperScript IV Reverse Transcriptase (RT) kit (Thermo Fisher Scientific, Waltham, MA, USA). All cDNA samples were diluted 3X with nuclease-free water and stored at −20 °C until further use. The gene expression quantification by RT-qPCR was performed with 4 μL of dilute cDNA and EvaGreen^®^ (Biotium, Fremont, CA, USA) dye in a final volume of 10 μL, according to the manufacturer’s instructions. The qRT-PCR reactions were performed using a LightCycler 480 from Roche^®^ (Mannheim, Germany). The fold change (FC) values of the qRT-PCR reactions were calculated using the Livak method [21], and statistical analyses were performed using R Studio version 4.1.0. 

Ten genes of *Phytophthora palmivora* and ten of *Arabidopsis thaliana* were analyzed. Genes of *P. palmivora* were selected based on previous analyses that showed key genes for the pathogenesis process [22], and the Arabidopsis genes were selected as hallmarks for plant defense response [23]. Primers for qRT-PCR analyses were designed using Primer3 (v.0.4.0) and are listed in Table 1. 

The relative biomass of *P. palmivora* was calculated using the Ppal*EF1a*/At*UBC9* genes ratio. The relative expression for each gene was calculated using Ppal*EF1a* and At*UBC9* genes as normalizers for *P. palmivora* and Arabidopsis, respectively. Controls for the calculation of ΔΔCt consisted of cDNA from axenically cultivated *P. palmivora* containing mycelia and sporangia (MY) and sterile water-inoculated leaves of Arabidopsis (Mock). Two technical replicates were analyzed for each of the three independent sample replicates at any given time point/treatment.

## 3. Results

### 3.1. Infection Dynamics of P. palmivora on Arabidopsis Col-0

**Figure 1:**
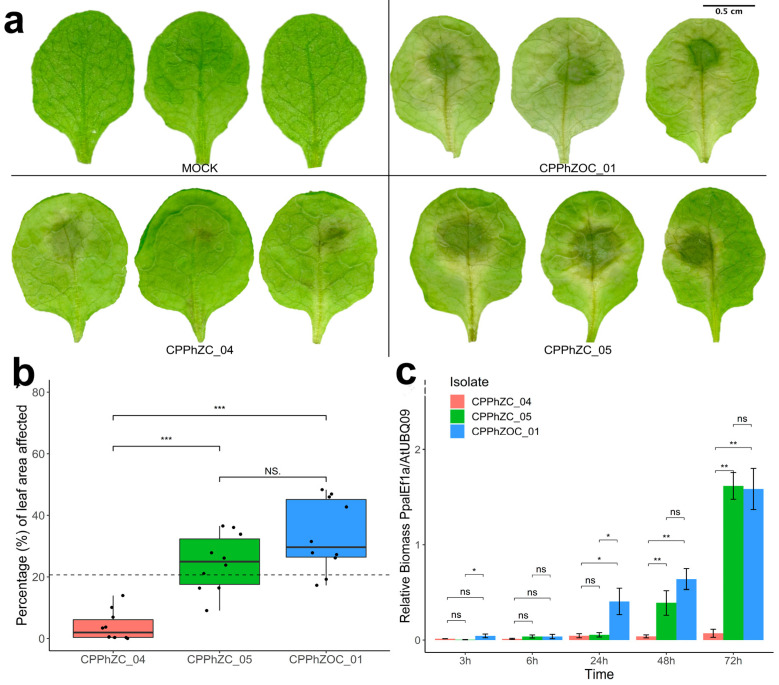
Characterization of the infection process of *P. palmivora* on detached Arabidopsis leaves. (**a**) Images of three representative leaves inoculated with *P. palmivora* isolates CPPhZOC_01, CPPhZOC_04, and CPPhZOC_05, with water (MOCK) used as a control. Leaves were scanned at 72 hpi. (**b**) Average percentage of leaf area affected by three *P. palmivora* isolates after 72 hpi. Each dot represents the average percentage of affected area from twelve leaves. The horizontal dotted line is the average across all the experiment. (**c**) Relative quantitative reverse transcription PCR (RT-qPCR) of pathogen biomass. The relative biomass of *P. palmivora* was calculated using specific primers targeting the *A. thaliana UBC9* gene (*AtUBC9*) and the *P. palmivora Ef1a* gene (*PpalEf1a*). Asterisks denote statistical significance based on the *t*-test (*** *p* < 0.001; ** *p* < 0.01; * *p* < 0.05; ns, not significant).

Three *P. palmivora* isolates were assessed for their infection capabilities on Arabidopsis Col-0. The isolates CPPhZC-05 and CPPhZOC-01 produced lesions covering 30% to 60% of the leaf area 72 h post-inoculation (Figure 1a). In contrast, CPPhZC-04 produced significantly smaller lesions, covering only 0% to 15% of the leaf area (Figure 1b). The biomass quantification of *P. palmivora* confirmed increased colonization over time for CPPhZC-05 and CPPhZOC-01, whereas CPPhZC-04 exhibited minimal growth (Figure 1c). These results show that visible lesions are correlated with pathogen development.

### 3.2. Root Inoculation and Symptom Evaluation

**Figure 2:**
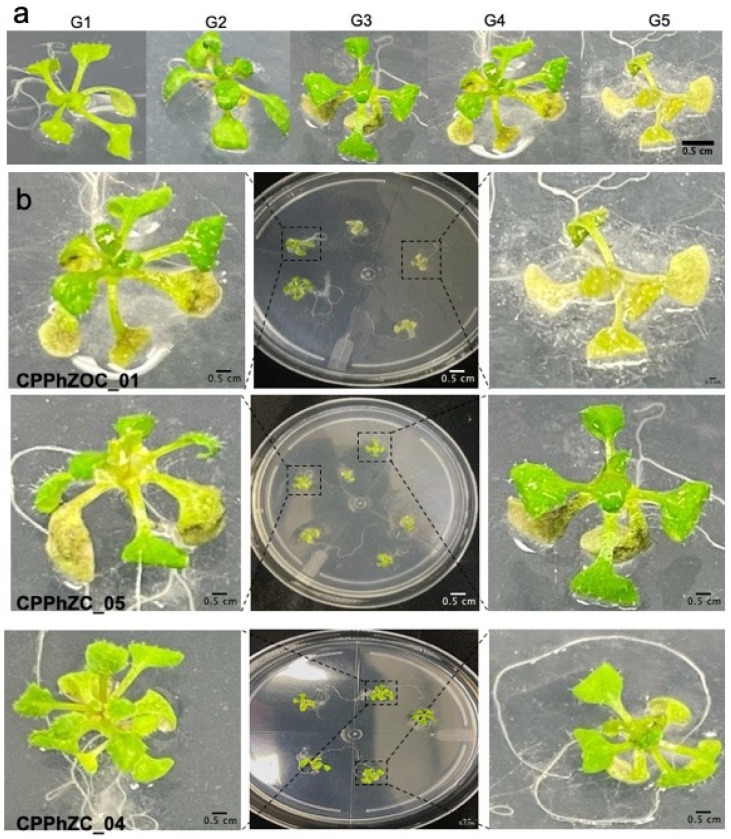
Whole-plant symptoms of Arabidopsis plants infected with *P. palmivora* isolates from oil palms with bud rot disease. (**a**) Severity disease scale of the infection of *P. palmivora* on seedlings of Arabidopsis. “G1”, healthy plants, “G2”, wilting and yellowing of the oldest leaves, “G3”, brown coloration of two leaves, “G4”, more than two brown colored leaves, “G5”, dead plants. (**b**) Inoculated seedlings with three isolates of *P. palmivora* on petri dishes. Roots of 10-day-old Col-0 seedlings were immersed in a zoospore suspension, and the disease symptoms were scored four days after inoculation. Scale bar = 0.5 cm.

*P. palmivora* is a soil-borne pathogen; thus, the roots of 10-day-old Col-0 seedlings were immersed in a zoospore suspension. Symptoms across the whole plant were observed three to four days post-inoculation, ranging from no visible symptoms to complete wilting. As a result, we categorized the disease severity on a scale: G1 (healthy plants), G2 (wilting and yellowing of the oldest leaves), G3 (brown coloration on two leaves), G4 (more than two brown-colored leaves), and G5 (dead plants) (Figure 2). 

**Figure 3:**
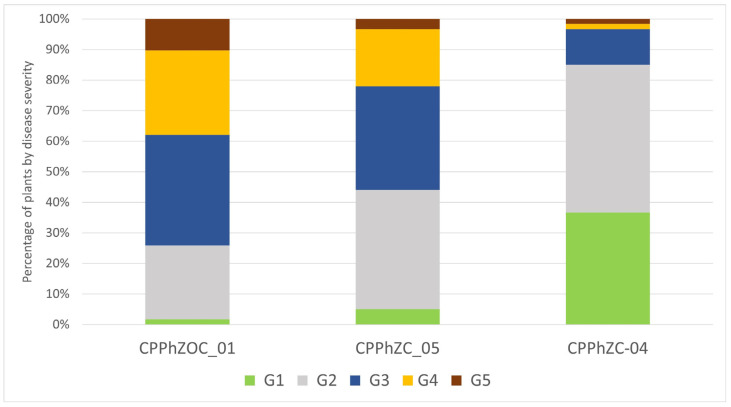
Percentage of Arabidopsis plants with different grades of disease severity after *P. palmivora* infection. Three isolates were used to inoculate 60 complete plants. “G1”, healthy plants; “G2”, wilting and yellowing of the oldest leaves; “G3”, brown coloration of two leaves; “G4”, more than two brown colored leaves; “G5”, dead plants. Roots of 10-day-old Col-0 seedlings were immersed in a zoospore suspension, and the disease symptoms were scored four days after inoculation.

The Arabidopsis plants exhibited different levels of disease severity after infection with *P. palmivora*. Around 60 to 70% of the plants inoculated with CPPhZC-05 and CPPhZOC-01 showed symptoms of wilting or complete collapse (G3, G4, G5) within four days (Figure 3), whereas 40% of the plants treated with CPPhZC-04 exhibited no symptoms or only mild symptoms (G1).

### 3.3. Microscopic Analysis of Infection Mechanisms

**Figure 4:**
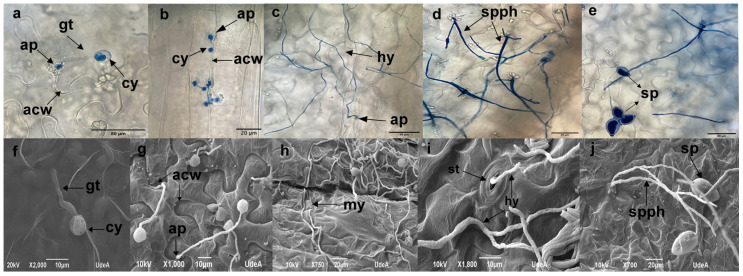
Compatible interaction between Arabidopsis and *Phytophthora palmivora*. Detached Arabidopsis leaves were inoculated with isolates ChPPZOC_01 and ChPPZC_05 from oil palms with bud rot disease. (**a**–**e**) Optical microscopy visualization. (**f**–**j**) Scanning electron microscopy. (**a**,**b**,**f**,**g**) Cyst with germ tube and appressorium generation penetrating the anticlinal cell wall junctions at 3 hpi and (**g**) 6 hpi. (**c**) Hyphae and (**h**) mycelium growing from the advancing edge of the lesion at 24 hpi and 48 hpi. (**d**). Sporangiophore emerging from tissue. (**i**) Hyphae emerging from stomata. (**e**–**j**) Ovoid sporangium at 72 hpi. Abbreviations: cyst (cy), germ tube (gt), appressorium (ap), anticlinal cell wall junctions (acw), Hyphae (hy), mycelium (my), stomata (st), sporangiophore (spph) and sporangium (sp).

Detailed microscopic observations of detached leaves using optical and SEM microscopy revealed that CPPhZC-05 and CPPhZOC-01 zoospores germinated and formed appressoria, penetrating the tissue at anticlinal cell wall junctions within 3 to 6 h post-inoculation (Figure 4a,b,f,g). During this biotrophic phase, frequent callose formations were observed at penetration sites (Figure 4b), although no macroscopic reactions were evident. By 24 and 48 h post-inoculation, the tissue showed extensive colonization by a dense network of hyphae (Figure 4c,h), and by 72 h, sporangiophores with ovoid sporangia had emerged from the tissue or stomata (Figure 4d,e,i,j), indicating that CPPhZC-05 and CPPhZOC-01 completed their life cycles in Arabidopsis.

**Figure 5:**
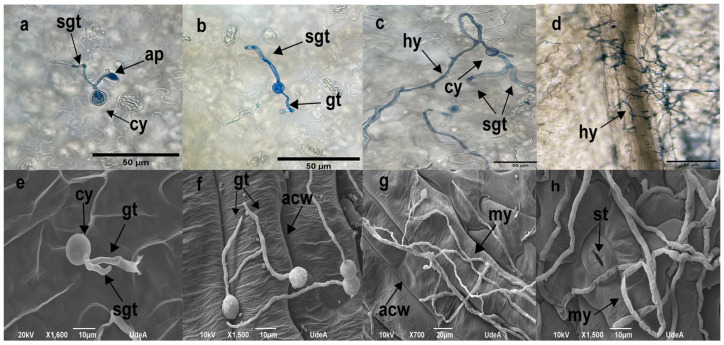
Non-compatible interaction between Arabidopsis and *Phytophthora palmivora*. Detached Arabidopsis leaves were inoculated with isolate ChPPZOC_04 from oil palm with bud rot disease. (**a**–**d**) Optical microscopy visualization. (**e**–**h**) Scanning electron microscopy. (**a**,**e**) Cyst with germ tube, secondary germ tube, and appressorium at 3 hpi. (**b**,**f**) Cyst without appressorium at 6 hpi. (**c**–**g**). Hyphal growth at the advancing edge without signs of tissue penetration and generation of secondary germ tubes at 24 hpi and 48 hpi. (**d**) Hyphal growth at 72 h, restricted to the inoculation site. (**h**) Stomata surrounded by mycelium at 72 hpi. Abbreviations: cyst (cy), germ tube (gt), second germ tube (sgt), appressorium (ap), anticlinal cell wall junctions (acw), hyphae (hy), mycelium (my), stomata (st).

In contrast, CPPhZC-04 demonstrated a reduced ability to penetrate tissue. Initial germ tube formation was observed between 3 and 6 h post-inoculation (Figure 5a,b,f), with continued growth on the leaf surface by 24 and 48 h to form hyphae (Figure 5c,g). By 72 h, although mycelium had developed, no sporangiophores or hyphae emerged from the tissue or stomata (Figure 5d,h), a behavior similar to other *Phytophthora* species which are unable to infect Arabidopsis or tobacco, as described in previous studies [11,13,24,25].

**Figure 6:**
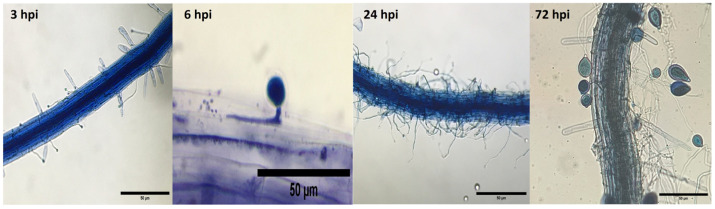
Colonization of Arabidopsis roots by *Phytophthora palmivora*. Roots of 10-day-old seedlings of Arabidopsis were inoculated with *P. palmivora* isolate CPPhZC-05 obtained from oil palms with bud rot disease. In this case, *P. palmivora* was able to grow inside the roots.

Furthermore, CPPhZOC_01 and CPPhZC_05 were able to grow internally, producing haustoria and sporangia (Figure 6), whereas CPPhZC_04 exhibited only vegetative growth with sporangia formation, failing to induce symptoms comparable to the other two isolates.

### 3.4. The Expression of Infection-Related Genes of P. palmivora Is Upregulated during Colonization of Arabidopsis Leaves

**Figure 7:**
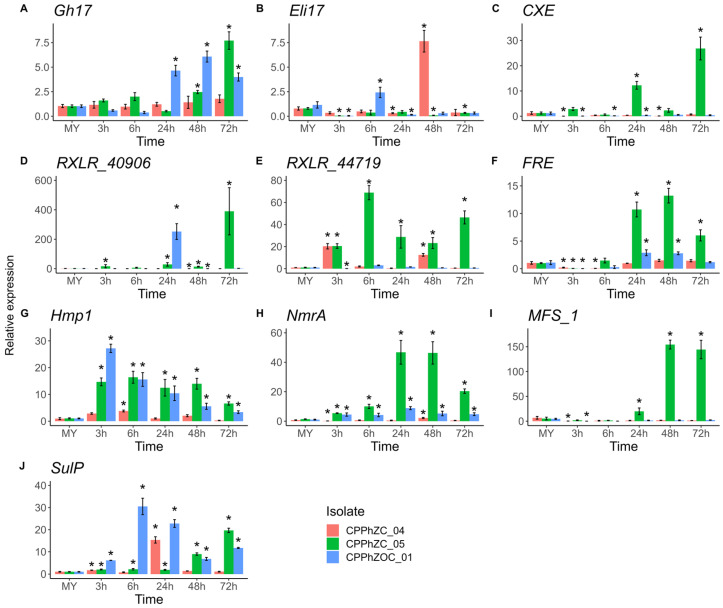
qRT-PCR analysis of *P. palmivora* infection-related genes during the colonization of detached Arabidopsis leaves. Gene expression was quantified relative to the *P. palmivora* reference gene *PpalEF1a*. (**A**) *Gh17*: Glycosyl hydrolase family 17. (**B**) *Eli17*: Elicitin, (**C**) *CXE*: Coesterase type *B.* (**D**,**E**) *RXLR* effector motive. (**F**) *FRE*: ferric reductase. (**G**) *Hmp1*: Haustorium-specific membrane protein. (**H**) *NmrA*: nitrogen metabolic regulation *A*. (**I**) *MFS_1*: major facilitator sugar transporter. (**J**) *SulP*: Sulfate Permease. The control group consisted of axenically cultivated *P. palmivora* (MY). Error bars represent standard errors from three biological replicates, and asterisks denote statistical significance between the control and each time point based on a two-tailed *t*-test (* *p* < 0.05).

Effectors are defined as molecules that manipulate cell structure and function. They are often assigned a dual function, facilitating infection or inducing the plant’s defense response [26]. Usually, the transcriptional induction of pathogen genes encoding secreted effector proteins is a hallmark of plant–pathogen interaction [27]. We examined the expression of five candidate effectors, two with hydrolytic domains (Gh17 (Glycosyl hydrolase family 17) and CXE (Coesterase type B)), an elicitin (hereafter called Eli17), and two RXLR motif effectors (RXLR_40906 and RXLR_44719). These are involved in oil palm colonization of the isolate CPPhZC_05. Additionally, we evaluated five other genes that included the transcription factor NmrA (nitrogen metabolic regulation A), transmembrane proteins such as Hmp1 (Haustorium-specific membrane protein), SulP (Sulfate Permease), MFS_1 (major facilitator, sugar transporter), and FRE (ferric reductase), which are co-expressed with candidate effectors during oil palm infection [22]. Our results show statistical differences in the expression of each gene compared to the controls (Figure 7). 

At the early stages, i.e., 3 and 6 h post-infection (hpi), the genes *Hmp1*, *NmrA*, *RXLR_44719*, and *SulP* were significantly upregulated compared to axenically cultured conditions. However, these expressions were variable among isolates. For instance, the isolate CPPhZC_04 only co-expressed *RXLR_44719* and *SulP* during this period, without any other genes. On the other hand, the isolate CPPhZOC_01 did not express the effector *RXLR_44719*; instead, it expressed Eli17. 

By 24 hpi, there were more notable changes in expression. In addition to the previously mentioned genes, there was also expression of genes such as *FRE*, *MFS_1*, *CXE*, *Gh17*, and RXLR_40906. At 48 h and 72 h, compatible isolates continued expressing FRE and MFS_1 genes, co-expressing with *Hmp1*, *NmrA*, and *Gh17*. 

The results point out that, during *P. palmivora* infection of Arabidopsis, the isolates CPPhZC_05 and CPPhZOC_01 secrete hydrolytic effectors and RXLR motif effectors, which are co-expressed with genes responsible for haustoria generation, nitrogen metabolism, and acquisition of nutrients such as sugars and sulfates. However, specific gene expression differs between the two compatible isolates, indicating that successful infection may occur through a distinct set of genes over time. 

Regarding the incompatible isolate CPPhZOC_04, it is important to note that the expression of *Hmp1*, associated with the germination of cysts, appressorium, and haustoria generation, remained low during the infection. This confirms the microscopic visualization results, where appressorium and tissue penetration were rarely seen. Only two effectors, RXLR_44719 and ELI17, were upregulated at 3 and 48 hpi, respectively.

### 3.5. Arabidopsis Induces Defense-Related Genes in Response to P. palmivora Infection

To determine whether Arabidopsis induces its defense system in response to *P. palmivora*, we examined genes of the salicylic acid (SA), jasmonic acid (JA), and ethylene (ET) pathways. These hormones are the main key players in plants’ innate immunity. We measured marker genes associated with each pathway through qRT-PCR. 

**Figure 8:**
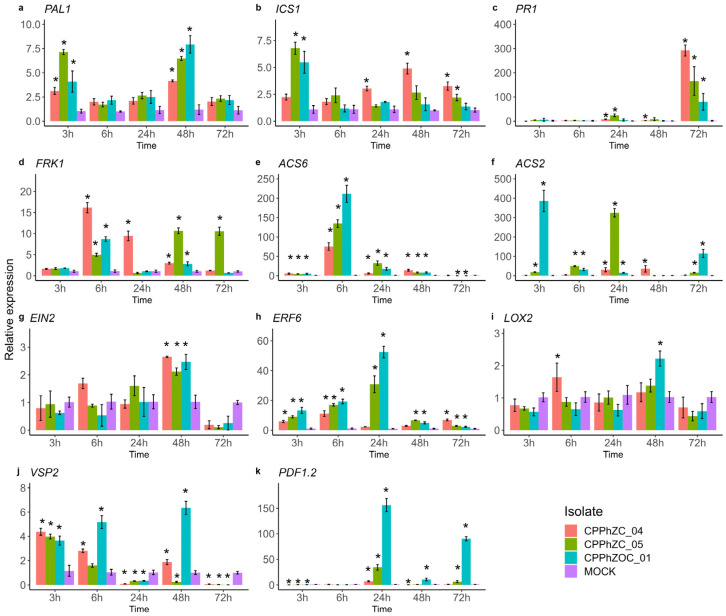
qRT-PCR analysis of *Arabidopsis* defense-related marker genes in response to *P. palmivora* colonization. (**a**) PAL1: Phenylalanine ammonia-lyase 1. (**b**) ICS1: Isochorismate synthase 1. (**c**) PR1: Pathogenesis-related protein 1. (**d**) FRK1: Flg22-induced receptor-like kinase 1. (**e**,**f**) ACS6, ACS2: 1-aminocyclopropane-1-carboxylic acid synthase. (**g**) EIN2: Ethylene-insensitive protein 2. (**h**) ERF6: Ethylene-responsive transcription factor 6. (**i**) LOX2: Lipoxygenase 2. (**j**) VSP2: Vegetative storage protein 2. (**k**) PDF1.2: plant defensing. Gene expression of each marker gene was quantified relative to the Arabidopsis reference gene *AtUBQ9*. The control group consisted of water inoculate leaves (MOCK) for each time point. Error bars represent standard errors from three biological replicates, and asterisks denote statistical significance between the control and each time point based on a two-tailed *t*-test (* *p* < 0.05).

*ICS1* (Isochorismate synthase1) and *PAL1* (Phenylalanine ammonia-lyase 1) are involved in the synthesis of SA [28], while *PR1* (Pathogenesis-related gene 1) is a well-established marker gene responsive to the accumulation of SA [29]. Arabidopsis upregulated both the *ISC1* and *PAL1* pathways in the presence of *P. palmivora*, indicating that AS synthesis occurred during infection (Figure 8a,b). All three isolates of *P. palmivora* induce AS biosynthesis; however, the compatible ones, CPPhZC_05 and CPPhZOC_01, induce a stronger response in ISC1 at the beginning of infection, followed by a decrease. However, in the presence of the incompatible isolate CPPhZC_04, ICS1 increases by 2.5-fold over time. PAL1 is also upregulated; however, it has been demonstrated that 98% of pathogen-induced SA in Arabidopsis is derived from the ICS1 pathway [28,30,31]. Hence, we assume that the major contributor to the synthesis of AS is ISC1 in the *P. palmivora*-Arabidopsis pathosystem. Typically, the accumulation of AS leads to the expression of PR1 [32], which correlates with the strong upregulation of PR1 generated by CPPhZC_04 at 72 hpi (Figure 8c). Also, PR1 proteins are secretory or vacuolar-target proteins with antimicrobial features [33,34]. 

The *FRK1* gene (Flg22-induced receptor-like kinase 1) is often used as a marker for PTI (pattern-triggered immunity). It activates downstream mitogen-activated protein kinase (MAPK) pathways after plant perception of PAMPs (pathogen-associated molecular patterns) [35]. Arabidopsis usually upregulates *FRK1* between 30 min and 8 h post-inoculation with a pathogen or peptide, fgl22 [36]. According to our findings, all three isolates caused upregulation of *FRK1* at 3 hpi (Figure 8d). Then, at 6 hpi, *FRK1* significantly increased in the presence of the isolate CPPhZC_04, but not in the presence of compatible isolates.

Another hormone involved in the plant defense response is ethylene (ET) [37]. To assess the production of ethylene (ET), we measured the expression levels of two genes: ACS2 and ACS6 (Figure 8e,f). These genes encode two different isoforms of 1-aminocyclopropane-1-carboxylic acid synthase, a crucial enzyme in the metabolic pathway for synthesizing ethylene. Our results showed that upregulation occurred between 3 and 24 hpi in the three isolates. However, the fold change was higher in the isolates CPPhZC_05 and CPPhZOC_01, indicating that ethylene is more abundantly produced when inoculating with these isolates. 

Our study showed that the gene *EIN2* was upregulated (Figure 8g). This gene plays a crucial role in the signaling pathway that activates the nucleus transcription complex of ET-responsive genes [38]. The gene *ERF6* encodes a transcriptional factor expressed when ethylene levels rise [39]. Our results showed that this gene was expressed between 3 and 24 hpi in the three isolates (Figure 8h). Once again, the CPPhZC_05 and CPPhZOC_01 isolates showed fold changes 5 to 20 times higher.

ERF6 is known to have a role in the positive regulation of jasmonic acid (JA) [39]. Therefore, its positive expression suggests that JA is produced due to infection of the three isolates of *P. palmivora*. To verify this, we quantified other markers involved in the synthesis of JA. *LOX2* (Lipoxygenase2) encodes a protein that is the main contributor to the synthesis of JA during leaf damage caused by wounds [40]. Although our results showed that its expression remained basal during infection compared to the Mock, in other photosystems, such as Arabidopsis-*P. parasitica*, this gene’s basal expression is sufficient to participate in JA synthesis [41]. 

Due to the accumulation of JA, a gene usually upregulated is *VSP2*, a vegetative storage protein is encoded [42]. This protein is a significant source of nutrients that accumulate in plant tissue under abiotic or biotic stress [43]. Our results showed upregulation after 3 hpi for the three isolates, then downregulation and raising at 48 hpi for CPPhZOC_01 and CPPhZC_04. 

Finally, the accumulation of JA led to the expression of the plant defensin gene *PDF1.2*, which was upregulated at 24 hpi and raised at 72 hpi. Interestingly, upregulation mainly occurred in the isolate CPPhZCO_01. 

These results suggest that *A. thaliana* triggers SA, JA, and ET biosynthesis during infection by *P. palmivora*. Furthermore, the resistance response to the CPPhZC_04 isolate might have been more closely associated with SA (PR*1* and *ICS1*), as there was a higher expression of genes associated with the SA pathway. In contrast, genes associated with JA and ET were more upregulated in the successful infection. Therefore, they seemed to mediate a susceptible response that facilitated infection by CPPhZC_05 and CPPhZOC_01.

## 4. Discussion

*Phytophthora palmivora* has many hosts and induces diseases in several crops. To broaden the alternatives in which *P. palmivora* can be studied, we examined its capacity to infect the well-known plant model Arabidopsis. Three oil palm—*P. palmivora*—isolates were used to infect detached leaves and whole seedlings of Arabidopsis Col-0.

Our findings demonstrate that two isolates can infect detached Arabidopsis Col-0 leaves or seedling roots following a 72 h cycle. Infection begins at the biotrophic phase, characterized by cyst germination, appressorium formation, and invasive hyphae formation, and progresses to the necrotrophic phase, where sporangia are generated. This life cycle is similar to other *Phytophthora* species capable of infecting Arabidopsis [12,14]. Additionally, an incompatible interaction was observed in the CPPhZC_04 isolate on detached leaves and whole seedlings. 

This variability provided an opportunity to increase our understanding of the differences among *P. palmivora* isolates collected from oil palms.

To study differences in isolates, we used a qRT-PCR approach to quantify ten previously detected *P. palmivora* genes involved in oil palm infection [22]. Hallmarks of the infection, *Hmp1* and *NmrA*, were expressed during compatible infection. Furthermore, the gene *MFS_1*, associated with a sugar transmembrane transporter, was detected mostly at 24 hpi, 48 hpi, and 72 hpi in the isolate CPPhZC_05 when hyphae were growing and spores were developing, which is consistent with the assumption that at those stages, filamentous microorganisms increase nutrient acquisition for development [44]. Likewise, sulfate permeases (SulP) are involved in the anabolism or removal of waste. In our results, a *SulP* gene changed dynamically through time and between the tree isolates. Similar patterns have been seen in *Phytophthora infestants* [44,45], where sulfur is likely to be used to synthesize cysteine, which is important for the production of antioxidants and secretion of effectors through the sulfurization pathway [46]. Furthermore, sulfate transport has been identified as one of the important processes that could be targeted for the control of disease caused by *P. infestants* in tomato plants [44]. 

Iron is another element linked to virulence in many pathogens, and is taken up from the host. Transporting iron to pathogens often involves ferric reductases that reduce ferric iron to assimilable ferrous [47]. According to our results, *P. palmivora* isolates CPPhZC_05 and CPPhZOC_01 upregulated the gene *FRE* at late stages, reaching a peak at 48 hpi. This suggests that iron was being taken up and that it might be important for *P. palmivora* growth in plants. In the Arabidopsis, *Phytophthora capsici*, pathosystem, iron depletion caused less infection in the plant. However, its relationship with virulence has not been proven [48]. 

Our result showed that genes related to the uptake of nutrients are co-expressed with candidate effectors and have similar pattern expressions as those previously reported when *P. palmivora* (CPPhZC_05) infected oil palm [22]. Specifically, we evaluated two genes that encode apoplastic effectors (*Gh17* and *CXE*), which contained hydrolytic domains. They were upregulated at 24 hpi when the pathogen invaded the tissue. This might suggest that *Gh17* and CXE play a role in degrading compounds of plant cell walls during *P. palmivora* infection. In the case of Gh17, a protein belonging to the glycosyl hydrolase family 17 has an O-Glycosyl hydrolyzing activity. In other pathosystems, such as *Cladosporium fulvum*-tomato, genes encoding Glycosyl hydrolase family-17 proteins are upregulated by the pathogen during the later phases of infection, when tomato leaves are necrotic, and the fungus is saprophytic [49].

On the other hand, CBX is a Carboxylesterase type B [50] of the carbohydrate esterase family 1 (CE1). It catalyzes ester bonds, breaks carbon-carbon bonds, and participates in decarboxylation [51]. In plants, it helps with growth, activates hormone signals, responds to biotic stress, and breaks down waxy polymers during germination [52,53,54,55,56]. It is found in hemibiotrophic lifestyle oomycetes and is upregulated in plant pathogenic oomycetes [57]. However, the precise role of Carboxylesterases during phytopathogen infection is still unknown. 

We also quantify *Eli17*, a secreted protein with an elicitin domain. Interestingly, according to our results, the gene *Eli17* was only upregulated by the incompatible isolate CPPhZC_04, while it was downregulated in the compatible ones. It is well known that elicitins are a family of small extracellular proteins with sterol-binding activity, which are found in oomycetes such as the *Phytophthora* and *Pythium* species [58]. Elicitins are characterized by their ability to induce a hypersensitive response (HR) in Solanaceae and Brassicaceae families when they infiltrate into leaves [59]. Although the Eli17 protein sequences of the three isolates are 98% identical (Figure S1), the high amount of the transcript expressed by CPPhZC_04 could explain part of the incompatibility. In other phytopathogens such as *P. parasitica* and *P. infestans*, the low production of elicitin correlates with increased virulence in *Nicotiana benthamiana* [60]. However, it is beyond this study’s scope to determine the level of recognition of this elicitin by Arabidopsis. 

In addition, the cytoplasmatic effectors (RXLR_40906 and RXLR_44719) were over-expressed, but the expression levels varied among isolates over time. Studies on expression profiles suggest that the regulation of RXLR effector expression is complex. It may be influenced by epigenetic mechanisms, such as RNA interference or histone methylation [61], or may be expressed depending on their functional activities; some are expressed early to suppress effector-triggered immunity (ETI), while others are expressed later to suppress pattern-triggered immunity (PTI) [62]. Therefore, these two genes may play distinct roles during infection. Concerning the response of Arabidopsis against *P. palmivora*, our findings indicate that SA, JA, and ET undergo regulation during the interaction. The induction of SA seems to be the primary resistance mechanism against *P. palmivora*. While the early induction of JA and ET impairs resistance, leading to a susceptibility state, it is widely known that a crosstalk between SA and JA signaling pathways occurs to adapt to infection by biotrophic and/or necrotrophic pathogens [63]. For instance, studies into the overexpression of the gene *AtRTP5* in Arabidopsis resulted in a higher accumulation of JA and lower SA production than wild-type plants (Col-0). Consequently, the mutants exhibited susceptibility to *P. parasitica* [41]. Hence, we hypothesize that Arabidopsis’ resistance against CPPhZC_04 is similar to what has been observed in *P. infestans* [24], *P. sojae* [62], and *P. parasitica* [12], where SA is produced in the initial stages to prevent infection. 

It is worth noting that we utilized detached leaves to quantify genes. Although we found similar susceptibilities in whole seedlings, we could not assert that the same molecular response would be generated in the roots. As reported in other studies, the resistance in roots differs from that in leaves [64,65,66,67].

Here, we provide initial insight into the interaction of *P. palmivora* and Arabidopsis Col-0. We found isolates with compatible and noncompatible interactions with Arabidopsis. We showed that detached Arabidopsis leaves are suitable for studying *P. palmivora* infection and the gene expression profiles of both organisms. The next steps involving this model will include transcriptomics and genomic analysis to elucidate differences in effector expression among the isolates of *P. palmivora* and Arabidopsis responses.

## Figures and Tables

**Table 1:** List of primers used for *P. palmivora* gene expression analysis by qRT-PCR.

Gen Name	Sequence (5′->3′)
*Gh17-Fw*	GGAGGCGTACTTCGGTCTGT
*Gh17-Rv*	GCCACCCACAACGGTGATTG
*Eli17-Fw*	GCGGAAGTGCCGTTCTATCC
*Eli17-Rv*	AGCCCATGGCCTGAGTATCG
*CXE-Fw*	CCTCGTGTGGATGGCTTTAT
*CXE-Rv*	CCTCGTGTAGCTCGTGTGAA
*RXLR_40906-Fw*	TCTCTTCCGGTGTCGATCCT
*RXLR_40906-Rv*	GAGCGATGTCATCCCACCAG
*RXLR_44719-Fw*	CTCGCTACGAAGTTGGCTGA
*RXLR_44719-Rv*	CAACATTGCGGTCCTTTGCA
*FRE-Fw*	GCGTTCGATTCTCAACGAGC
*FRE-Rv*	CTAGCATCGTCACGGCAGAT
*Hpm1-Fw*	TGCCATTCTTGATCTGCCGT
*Hpm1-Rv*	CAGATTCACGCAGCATGAGC
*NmrA-Fv*	CAACGTGGTTGTTCGGTGAC
*NmrA-Rv*	AGGCAGGAATGGGATCTCCT
*MFS_1-Fw*	CTGGGTGAGTCTCCTCGGTA
*MFS_1-Rv*	GCCACGTAGTAACCGGGTAG
*SulP-Fw*	TTGCCATCTTCCTCATGCGT
*SulP-Rv*	CGAAGCGGTCACCATCGATA

## Data Availability

The raw data supporting the conclusions of this article will be made available by the authors on request.

## Supplementary Materials

The following supporting information can be downloaded at: https://www.mdpi.com/article/10.3390/jof10070446/s1, Figure S1: Protein alignment of RXLR effectors and ELI17.
