# Supplementary figures and images for: Infection of Phytophthora palmivora Isolates on Arabidopsis thaliana
Source: J Fungi (Basel). 2024 Jun 26;10(7):446. doi: 10.3390/jof10070446 (PMC11277810; doi:10.3390/jof10070446)

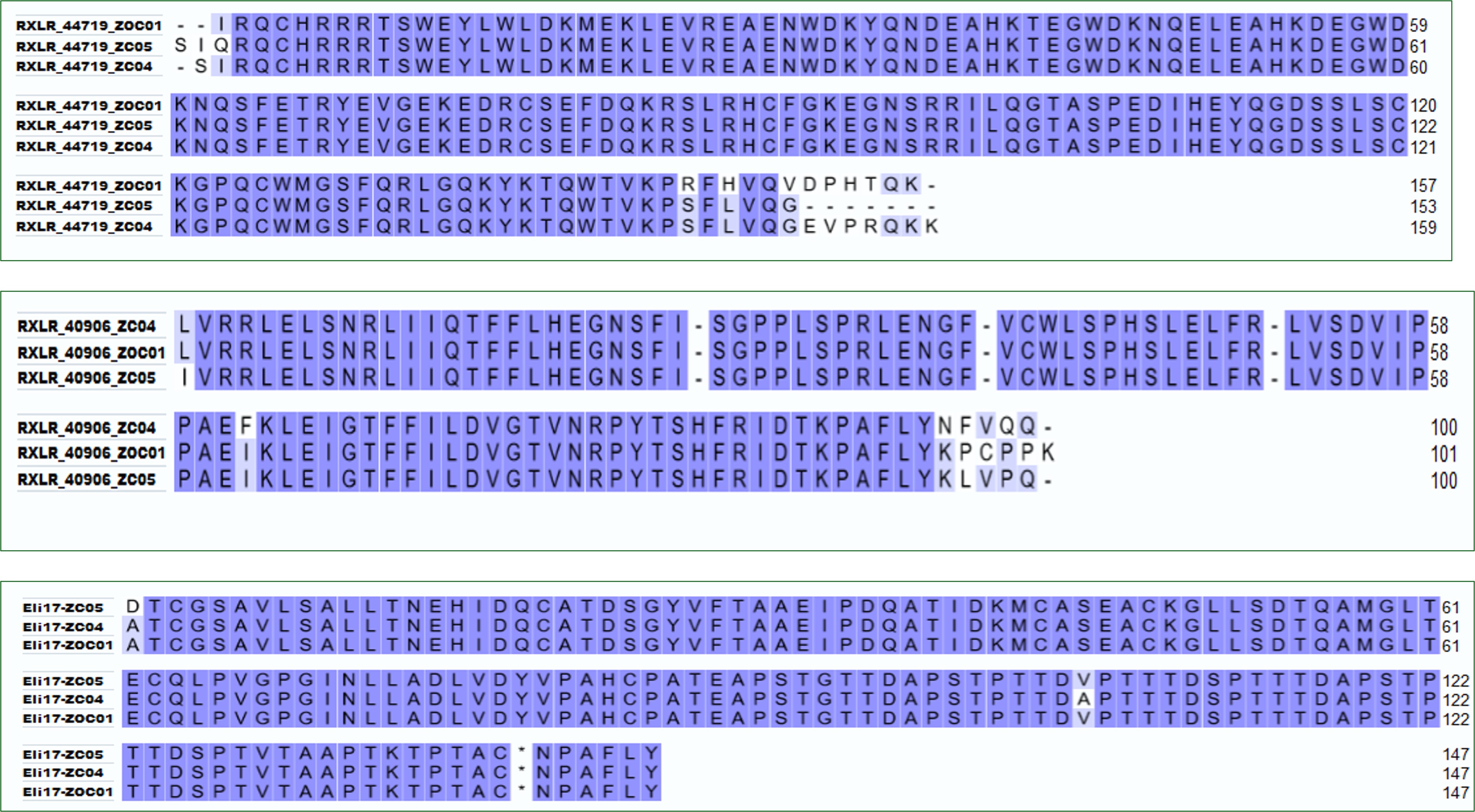

Supplement: Supplementary file 1 [file jof-10-00446-s001.zip › Figure S1. Protein alignment of RXLR effectors and ELI17.jpg]
